# *FADS* Genetic Variants in Taiwanese Modify Association of DHA Intake and Its Proportions in Human Milk

**DOI:** 10.3390/nu12020543

**Published:** 2020-02-20

**Authors:** Wen-Chieh Wu, Hung-Chih Lin, Wen-Ling Liao, Yueh-Ying Tsai, An-Chyi Chen, Hsiang-Chun Chen, Hsiang-Yu Lin, Li-Na Liao, Pei-Min Chao

**Affiliations:** 1 PhD Program for Health Science and Industry, China Medical University, Taichung 404, Taiwan; willson60805@gmail.com; 2 Division of Neonatology, Children’s Hospital, China Medical University, Taichung 404, Taiwan; d0373.cmuh@gmail.com (H.-C.L.); hylin.neo@gmail.com (H.-Y.L.); 3 Asia University Hospital, Asia University, Taichung 413, Taiwan; 4 School of Chinese Medicine, China Medical University, Taichung 404, Taiwan; 5 Graduate Institute of Integrated Medicine, China Medical University, Taichung 404, Taiwan; wl0129@mail.cmu.edu.tw; 6 Center for Personalized Medicine, China Medical University Hospital, Taichung 404, Taiwan; 7 Phalanx Biotech Group, Inc., Hsinchu 300, Taiwan; darrentsai@phalanxbiotech.com; 8 Division of Pediatric Hepatology and Gastroenterology, Children’s Hospital, China Medical University, Taichung 404, Taiwan; d8427@mail.cmuh.org.tw; 9 College of Medicine, China Medical University, Taichung 404, Taiwan; 10 Chen Hsiang-Chun Postpartum Nursing Home, Tainan 701, Taiwan; a810383@yahoo.com.tw; 11 Department of Public Health, China Medical University, Taichung 404, Taiwan; 12 Department of Nutrition, China Medical University, Taichung 404, Taiwan

**Keywords:** DHA, fish, single nucleotide polymorphism, *FADS* gene, human milk

## Abstract

Our objective was to determine how docosahexaenoic acid (DHA) proportions in human milk are modulated by maternal *FADS* gene variants and dietary intake in Taiwanese women. Inclusion criteria included being healthy, 20–40 y old, having had a full-term baby that they intended to breast feed for at least 1 month, and willingness to participate in this study. Intake of DHA was assessed by food frequency questionnaire and fatty acids were analyzed in human milk samples collected 3–4 weeks postpartum. Based on multiple linear regression of data from 164 mothers that completed this study, there was 0.28% (FA%) reduction in milk DHA in high versus low genetic risk (stratified by whether minor allele numbers were ≥ 3 in rs1535 and rs174448) and 0.45% reduction in low versus high intake (stratified by whether DHA intake reached 200 mg/d). There was a significant gene–diet interaction; mothers with low genetic risk only had high milk DHA proportions with high DHA intake, whereas for mothers with high genetic risk, dietary effects were quite limited. Therefore, for *FADS* single nucleotide polymorphism in Taiwanese women, increasing DHA intake did not correct low milk DHA proportions in those with a high-risk genotype. Diet only conferred benefits to those with a low-risk genotype. Trial registration: This trial was retrospectively registered (Feb 12, 2019) in ClinicalTrials.gov (No. NCT03842891, https://clinicaltrials.gov/ct2/show/NCT03842891).

## 1. Introduction 

Docosahexaenoic acid (DHA, 22:6n-3) is a main fatty acid component of structural lipids of cell membranes, particularly those of brain gray matter and retina [1]. There is substantial accretion of DHA in the human brain, primarily from the last trimester of pregnancy to 2 years old [2]. Based on animal studies, DHA deficiency during pregnancy and lactation caused irreversible long-term damage to the central nervous system [3,4,5]. Despite a lack of conclusive evidence, the advantages of infants consuming human milk versus formula, including enhanced neurological and visual maturation and reduced allergies, are generally attributed to the presence of long-chain polyunsaturated fatty acids (LCPUFA) in human milk [6]. To mimic fatty acid profiles of erythrocytes in breast-fed infants, many manufacturers now include arachidonic acid (AA, 20:4n-6) and DHA in formula for term babies. 

Exclusive breastfeeding for first 6 months of life is promoted by many countries and strongly recommended by the World Health Organization (WHO) and others [7]. Although DHA in human milk is critical for infant development, proportions vary dramatically among individuals or across countries [8]. In human milk, DHA comprises 0.06%–1.4% of total fatty acid content; proportions are highest among coastal populations, lowest in populations distant from the coast, and directly related to dietary intake, including marine food and DHA supplements [9]. The Early Nutrition Academy recommends pregnant and lactating women consume 200 mg of DHA daily [10]. Despite no consensus regarding optimal DHA proportions in human milk, infants receiving human milk with > 0.32% DHA apparently had better visual and neural system development than those consuming milk with ≤ 0.2% DHA [9,11,12]. 

In addition to dietary intake, genetic variants are associated with altered n-6 and n-3 LCPUFA status [13,14,15]. Most studies have focused on *FADS1* and *FADS2.* They encode fatty acid Δ5 desaturase (D5D) and Δ6 desaturase (D6D), respectively, which are involved in conversion of dietary precursors linoleic acid and α-linolenic acid into n-6 and n-3 LCPUFA. Three studies in Caucasians [16,17,18] and one from China [19] demonstrated that the genotype of the *FADS* gene cluster influences the fatty acid composition of human milk. Among Caucasian and European people, minor allele homozygotes of rs174553 (linked with rs99780 and rs174583) and rs174575 [16], or rs3834458 (linked with rs1535), rs174561, and rs174575 single nucleotide polymorphisms (SNPs) [17] had more 18C precursors but less AA, eicosapentaenoic acid (EPA), docosapentaenoic acid (DPA), and DHA in blood phospholipids and milk of pregnant and lactating women. Likewise, Han Chinese women also had lower γ-linolenic acid (GLA) and AA concentrations in their breast milk, implying lower D5D and D6D activity, in minor allele carriers of *FADS1/2* SNPs and haplotypes (rs1535, rs3834458-rs1535, rs1535-rs174575, and rs174547-rs174553), whereas there was no effect on DHA [19]. For minor allele homozygotes, higher DHA intake compensated for lower DHA in women’s plasma phospholipids but not in their milk [17]. Consequently, genotype modified dietary effects on DHA status in breast milk.

Although human milk is regarded as a “gold standard” food for infants, its nutritional quality is not always guaranteed, because of highly variable DHA proportions. Our objective was to determine the effects of dietary DHA intake and *FADS* SNP on human milk DHA proportions in Taiwanese women, to inform personalized dietary guides for pregnant and lactating women.

## 2. Materials and Methods 

### 2.1. Study Design and Subjects Recruitment

In Taiwan, most women choose to stay in postpartum care centers for approximately 1 to 6 weeks after delivery. Therefore, from September 2017 to September 2018, we recruited participants from three postpartum care centers in Taichung and Tainan, Taiwan. Trained nurses explained the purpose of our study and assisted in completing questionnaires and collecting samples. Inclusion criteria were being Taiwanese, 20–40 y of age, healthy, having a full-term baby (≥ 36 wk of gestation), and planning to breastfeed for at least 1 month. Women who met all inclusion criteria were enrolled if they voluntarily gave written informed consent. Questionnaires for personal data and DHA intake, as well as oral swabs (for genotyping), were completed in postpartum care centers. Breast milk samples were collected at 3–4 wk postpartum, either at postpartum centers or home (depending on duration of stay), kept at 0 °C, and express-delivered to our laboratory. This time point was selected because: (1) the composition is stable (i.e., mature milk); and (2) a previous study indicated the genetic effect on human milk fatty acid composition might be more pronounced at early stages [18]. This study was conducted according to Declaration of Helsinki guidelines. All procedures involving human subjects were approved by the Research Ethics Committee of China Medical University and Hospital, Taichung, Taiwan (CMUH106-REC1-115), and the study was retrospectively (February 12, 2019) registered on ClinicalTrials.gov (NCT03842891).

### 2.2. Selection of SNPs

Four SNPs from the *FADS* gene cluster were selected: SNP rs1535 and rs174575 in the *FADS2* gene, SNP rs174448 located in the intergenic region between the *FADS2* and *FADS3* genes, as well as SNP rs174561 in the *FADS1* gene. These four SNPs are significantly associated with concentrations of polyunsaturated fatty acids (PUFAs) in erythrocytes or plasma, and fulfill the requirement of minor allele frequency (MAF) ≥ 10% in Taiwan BioBank. The four SNPs selected are not in complete linkage disequilibrium with each other.

### 2.3. DNA Extraction and SNP Genotyping

DNA was extracted from oral swabs by Phalanx Service Laboratory using the MagPurix Forensic DNA Extraction Kit (Zinexts Life Science, New Taipei City, Taiwan). DNA yield and purity were determined by NanoDrop 1000 (Thermo Fisher Scientific, Carlsbad, CA, USA). The four *FADS* SNPs were genotyped with TaqMan SNP Genotyping assays. For real-time polymerase chain reaction(PCR), mixtures of iTaq SYBR^®^ Green supermix (Bio-Rad, Hercules, CA, USA), primers, and probes (Table S1) were incubated at 95 °C for 3 min, followed by amplification using 39 cycles of 2 steps (95 °C for 10 s, 60 °C for 10 s, and 72 °C for 1 min) on an Applied Biosystems (ABI) Prism 7900HT sequence detection system.

### 2.4. Dietary Information

Information of DHA intake during the peripartum period was collected with a food frequency questionnaire (FFQ). Based on food items primarily contributing to dietary DHA intake in Taiwan (Nutrition and Health Survey in Taiwan), consumption frequency and portion size of fish (milk fish, pacific saury, Greenland halibut, salmon, mackerel, tilapia, tuna, and sea perch) over the previous month were answered by participants. In addition to foods, dietary supplementation of fish oil, algae oil, or functional foods containing DHA were recorded (brand and amount). Total DHA intake from food and supplements was calculated.

### 2.5. Milk Collection and Fatty Acid Analysis

Mothers were instructed to collect a milk sample (5–10 mL) in the morning before breast feeding their baby from the second breast (after finishing the first). On the day of collection, milk storage bags were kept at 0 °C, express-delivered to our laboratory, and stored at -80 °C until analysis. To avoid potential loss of medium-chain fatty acids, milk was thawed in ice-cold water and directly transmethylated without lipid extraction [20]. Briefly, 100 μL milk reacted with 2.5 mL NaOCH_3_/MeOH solution (0.5 M) at 80 °C for 30 min, and then after cooling, 2.5 mL BF_3_/MeOH (14%) was added and allowed to react at 80 °C for 3 min. Fatty acid methyl esters generated were dissolved in hexane for fatty acid analysis in a Hewlett-Packard 5890 gas chromatograph using flame ionization detection on a DB-1 fused silica capillary column (60 m × 0.25 mm × 0.1 um; Agilent, Inc, Palo Alto, CA, USA) with nitrogen as carrier gas (1.5 ml/min). The oven temperature program was set at 60 °C for 2 min, then increased at 10 °C/min to 170 °C, then at 3 °C/min to 270 °C, and held at 270 °C for 15 min. Fatty acid peaks were identified by comparison to retention times with authentic standards. 

### 2.6. Statistical Analyses

Study of individuals’ characteristics, including age, body mass index (BMI), consumption of fish, DHA supplements, and total DHA intake, are presented as the mean and standard deviation or proportions for continuous and categorical variables, respectively. The departure of the Hardy–Weinberg equilibrium (HWE) for each SNP was analyzed with PLINK (v1.07) [21]. One-way analysis of variance (ANOVA) was used to detect differences between means of fatty acid compositions of human milk in three genotypes for each SNP. To define a person’s individual genetic risk for human milk DHA, simple linear regression analysis was done for each SNP and a genetic risk score for each individual was determined by summing the number of minor alleles (coded as 0, 1, 2, 3, and 4) that they had. Low and high genetic risks were defined as scores of ≥ 3 and ≤ 2, respectively. To assess the association of human milk DHA proportions with genetic risk, total DHA intake, and their interaction, multiple linear regression analysis was done, which was adjusted for age, BMI, parity (multiparous vs. primiparous), and gestational weeks. The gene–diet interaction was examined by entering the product term of genetic risk (high vs. low) and total DHA intake (low vs. high). Next, from the multiple linear regression model, we calculated these adjusted means and their 95% confidence intervals (CIs) of human milk DHA among four groups of genetic risk and total DHA intake statuses. The significance level was set as a two-sided *p*-value < 0.05. All statistical analyses used Statistics Analysis System (SAS) software, Version 9.4 (SAS Institute Inc., Cary, NC, USA).

## 3. Results

In total, 191 women were recruited. After exclusions due to failures in sample collection or missing data in questionnaires, 164 women were included for data analysis. Their basal characteristics are shown (Table 1). Fish consumption averaged 4.5 servings per week and approximately one-third of women consumed DHA supplements during pregnancy and lactation. Total DHA intake was 143 ± 126 mg/d (range, 0–457 and median 93), with only 27% reaching the recommended intake (200 mg/d). 

Although four SNPs were genotyped with a success rate of 100% and confirmed to be polymorphic, SNPs rs174575 and rs174561 were not in HWE (*P* < 0.05) in our study population; therefore, further analyses of these two SNPs related with human milk DHA were not conducted. The other two SNPs rs1535 and rs174448 were consistent with HWE and their minor allele frequencies (MAFs) were 44.5% and 24.1%, in line with Taiwan Biobank data and southern Han Chinese in 1000 Genomes data (Table 2). Note that rs1535 G rather than A are minor alleles in other populations (see Discussion). 

The fatty acid composition of human milk stratified by genotypes is shown (Table 3). Neither rs1535 nor rs174448 genotype affected fatty acid composition, except for 20:1n-9 (*P* < 0.05), which was increased by the minor allele of rs1535. However, when the total number of minor alleles from both rs1535 and rs174448 were considered against breast milk DHA proportions, there was a protective effect of minor alleles; milk DHA proportions were significantly higher in mothers with minor allele number of 3 (DHA%: 0.47 ± 0.45) compared to those with minor allele number of 0, 1, or 2 (DHA%: 0.38 ± 0.23). There was no participant in our study carrying four minor alleles. Therefore, mothers with minor allele numbers ≤ 2 were designated high genetic risk, whereas mothers with minor allele number 3 were designated low genetic risk. Regarding dietary effect, there was a positive correlation between total DHA intake and human milk DHA (r = 0.18, *p*< 0.05; data not shown).

**Figure 1:**
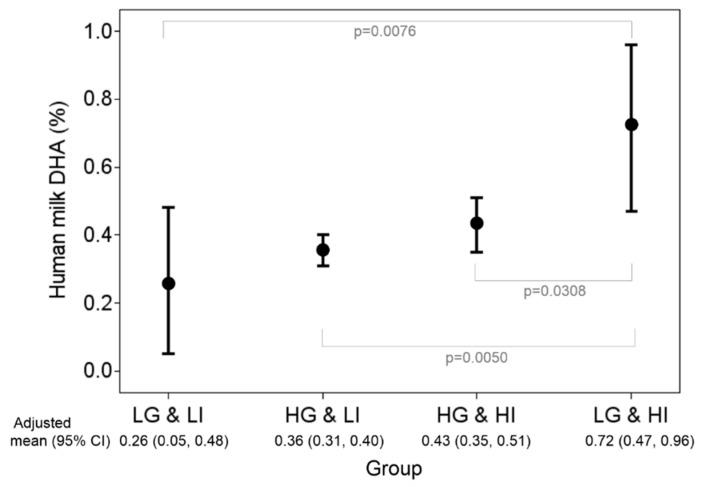
Stratified analysis by groups of genetic risk and total DHA intake statuses. Adjusted means and their 95% CIs of human milk DHA were obtained from the multiple linear regression model with consideration of age, body mass index , parity, and duration of pregnancy. LG: low genetic risk (minor allele number = 3 in rs1535 and rs174448); HG: high genetic risk (minor allele number ≤ 2 in rs1535 and rs174448); LI: low DHA intake (< 200 mg/d); HI: high DHA intake (≥ 200 mg/d).

Based on multiple linear regression, the main factors affecting milk DHA proportions were genotypes and DHA intake (Table 4); there was a 0.28% reduction in milk DHA in high versus low genetic risk and 0.45% reduction in low versus high DHA intake (< 200 vs. ≥ 200 mg/d), with a significant gene–diet interaction. There were no significant effects of age, BMI, parity, or gestational age on milk DHA proportions. Accordingly, participants were stratified according to high or low genetic risk and DHA intake (Figure 1). Milk DHA proportions were highest in mothers with low genetic risk and high DHA intake. However, for mothers with high genetic risk, dietary effects (total DHA intake) were quite limited. 

## 4. Discussion

For human milk DHA proportions, in addition to main effects of diet and genotype, there was a significant gene–diet interaction, consistent with the KOALA birth cohort report from the Netherlands [17]. In that study, maternal *FADS* genotypes (rs174561, 174575, and rs3834458, which is highly correlated with rs1535 [22]) and fatty fish intake were investigated for two outcomes (i.e., DHA proportions in erythrocyte plasma phospholipids and in breast milk (36 wk of pregnancy and 1 month postpartum, respectively). Although plasma DHA proportions increased in proportion to fish and fish oil intake irrespective of genotype, milk DHA proportions increased with increasing fish and fish oil intake only in specific genotypes (i.e., major allele carriers). The present study confirmed that low milk DHA proportions in mothers with a high-risk genotype could not be overcome by increasing DHA dietary intake, although high dietary DHA intake enhanced milk DNA proportions in women with a low-risk genotype (gene–diet interaction). Moreover, similar to a study conducted in south Germany (Ulm), where a relatively low amount of sea fish is consumed [18], we find genetic variants have little effect on human milk DHA for mothers with low DHA intake.

Human milk DHA comes from dietary preformed DHA (i.e., exogenous source) or produced from α-linolenic acid via *FADS1/2* and *ELOVL2/5* in liver or mammary glands (i.e., endogenous synthesis). Variants of *FADS* and *ELOVL* altered fatty acid profiles by increasing 18C substrates while decreasing both n-6 and n-3 LCPUFA products; therefore, a decreased gene transcription rate or enzyme activity was proposed. Moreover, minor allele carriers of *FADS* are generally associated with lower DHA proportions in serum, erythrocyte membranes, and breast milk [23]. Although both diet and genetics contribute to human milk DHA proportions, the interaction between diet and genetics is difficult to understand. The notion that the *FADS* gene cluster could be linked to n-3 LCPUFA transportation or incorporation into milk was proposed [17] but has not been substantiated. 

An ethnic difference was noticed in rs1535 SNP. The minor/major allele reported in Caucasian and Asian studies was G/A [19,24], but was A/G in our study population. The A allele frequency of rs1535 in our study (44.5%) was close to TW Biobank (43.2%) and CHS Southern Han Chinese (41.4%) results. However, the A allele frequency in other Asians, such as CHB Han Chinese in Beijing and JPT Japanese in Tokyo, Japan, as well as in Caucasians, is > 50%. Taiwanese people are 80% southern Han Chinese (sharing ancestors with persons in Guangdong, Guangxi, Fujian, Hong Kong, Macau, and Hainan) [25]. The genetic distinction between northern and southern Han Chinese has been recognized [26]. Nevertheless, with opposite major/minor frequency, this study agreed with others that GG homozygotes of rs1535 are disadvantageous for tissue DHA [15,17,24], meaning the G allele (major allele in this study, but minor in others) tended to decrease milk DHA proportions (Table 3). A similar example is *FADS1* rs174547; the major allele for this SNP differed between Caucasians (T) and Asians (C). Carriers of the C allele consistently have lower desaturase activity than carriers of the T allele in both populations [27]. 

SNP rs1535 is located on *FADS2* (encoding D6D), a rate-limiting enzyme for endogenous synthesis of LCPUFA. It is intriguing to ask why a disadvantageous variant was dominant in southern Han Chinese. Perhaps alleviating dependence on endogenous synthesis by accessibility of fish (DHA) intake in southern (relative to northern) Han Chinese has a role.

In this study, a greater accumulated minor allele number (A in rs1535 and G in rs174448) of *FADS* SNP favored higher milk DHA proportions. In contrast, in a Danish study of DHA status on infants’ genotypes and diet, G in rs1535 and A in rs174448 were DHA-increasing alleles [22]. Perhaps *FADS* isoforms regulate endogenous LCPUFA biosynthesis in a manner specific to tissue, organelle, and developmental stage [28], therefore the SNP effect on transcription rate or enzyme activity may differ between infants and adults.

Human milk DHA proportion in the present study seemed lower than previous reports from Taiwan [29,30] (0.39 vs. 0.8–1%), irrespective of the postpartum period. This may be partly attributed to the methodological issues, since we used no extraction, thus preventing loss of medium-chain fatty acids [16]; otherwise, DHA proportions could be artificially elevated. Due to methodological variations, a comparison of DHA proportions in human milk across countries is difficult. A nine-country comparison employing the same collection and analysis methods confirmed coastal countries (Japan, Philippines, and Chile) had human milk DHA ≥ 0.4%, which was superior to interior parts of Canada and the US (0.2%) [31]. The mean value in this study approached coastal countries and was slightly higher than the global average (0.32%) [8]. Compared to most formula having AA:DHA ratios between 1.5:1 and 2:1, Taiwanese women have a milk AA:DHA ratio of 1:1, which is again an intermediate value between Japan (0.5:1) and the US (3.16:1) [31]. Substantial variation in DHA proportions in human milk (i.e., a 26-fold individual variation (0.06–1.65%)) was also observed in this study. Moreover, the high linoleic acid (18:2 n-6) proportions and n6/n3 ratio in Taiwanese human milk observed in this study and others [29,30] raised concerns of overusing n-6 vegetable oil as cooking oil (sunflower oil, soybean oil) in Taiwan, since a high n6/n3 ratio in human milk may be linked to immune and inflammatory diseases in children [9,32].

It has been suggested that proportions of human milk DHA should be > 0.32% to support 70 mg DHA/d accretion in the forebrain of babies and a linear pathway through 1 y of age [9,33]. In addition, nursing babies consuming breast milk with < 0.2% DHA had less-developed visual acuity and cognition compared to babies consuming breast milk with > 0.32% DHA [9,11]. In this study, < 15% mothers had milk DHA < 0.2%, mainly attributed to inadequate DHA intake, as 80% consumed below the recommended DHA intake. For pregnant or lactating women, adequate DHA intake is always encouraged. Although this study replicated a report [17] that a high DHA intake did not ensure high proportions in breast milk (particularly for mothers with high genetic risk), a randomized clinical trial should be conducted in mothers with varying genotypes to determine effects of DHA supplementation on milk DHA status.

According to a nutritional adequacy hypothesis, once an individual’s nutritional requirement is met, further intake of the given nutrient yields no additional benefit [34]. In that regard, there is no convincing evidence that milk DHA > 0.32% yields further benefits [35]. We believe efforts should be focused on mothers with milk DHA < 0.2%. In addition to encouraging DHA intake in pregnant and lactating mothers, timely introduction of complementary foods such as DHA sources for infants should be helpful. 

DHA is an important component of membranes in brain and retinal cells. Studies in animal models and humans suggested that adequate DHA is important for cognitive development through several processes, including biogenesis and fluidity of cellular membranes, neurogenesis, neurotransmission, and protection against oxidative stress [36,37]. Although breastfeeding is regarded as increasing child IQ due to increased DHA intake, Capsi et al. [38] and Steer et al. [39] reported opposite results in child genotypes of *FADS* SNPs (rs174575 and rs1535), who had better IQ results if breastfed. Morales et al. demonstrated maternal genotypes with a high *FADS2* (D6D) activity corresponded to a low *FADS1* (D5D) activity, and contributed to greater colostrum EPA and DHA that appeared to be crucial for child cognition at 14 months of age, whereas this association was altered by children’s genotypes [40]. Results of our study cannot be extrapolated to predict for mothers with specific genotypes who may impart the greatest benefits in terms of increasing their child’s IQ by breastfeeding. There is accumulating evidence that breastfeeding favoring child IQ is related not only to maternal *FADS* genotypes, but also the child’s own genotypes, complementary feeding with DHA sources, and breastfeeding duration [34]. 

This is apparently the first study to investigate the gene–diet interaction in human milk DHA in Asians, with relevance particularly for southern Han Chinese. Nevertheless, a limitation of this study is that only two *FADS* SNPs (rs1535 and rs174448) were used, as the genotype distribution of rs174575 and rs174561 deviated from HWE. In the future, cohorts with larger sample sizes and broader *FADS* clusters are needed, so that haplotype construction and analyses can be conducted to delineate this gene–diet interaction on human milk DHA concentration. In addition, people may criticize the representative sample, as only a small amount (5–10 mL) of milk sample was taken. We admit a 24-hour milk collection (pooled samples from each feeding) is better, but this will increase the difficulty in recruiting participants. Another limitation is FFQ may not have accurately assessed DHA intake. Furthermore, all mothers in this study followed traditions of Chinese medicine to promote postpartum recuperation; for example, for ~ 1 month postpartum, their dietary intake of selecting hot-/warm-natured food (i.e., improves the circulation and increases “qi” or energy of our body) but avoiding cold-/cooling-natured foods (i.e., reduces heat whereas may cause diarrhea) may not be representative of their usual diet. However, fish are generally regarded as neutral or warm in nature and dietary habits of eating fish appear to be relatively stable [9]. Therefore, the DHA intake data obtained here may not have been completely accurate, but distinguishable for high versus low intake. 

Lastly, we would like to indicate that although this study showed that DHA intake did not improve milk DHA in mothers with disadvantage genotypes, we could not exclude the possibility that a high dose supplementation (1000 mg DHA/d) might be able to overcome this genetic barrier. Milk DHA has been demonstrated to be substantially increased by supplementation of this high dose (but not the recommended 200 mg/day dose) in human milk donors [41,42], which benefits extremely preterm infants (< 28 weeks gestational age) who miss DHA accretion during the third trimester of development [42].

## 5. Conclusions

In contrast to Caucasians and Han Chinese, G instead of A is a major allele of rs1535 in Taiwanese. In the present study, Taiwanese mothers with high genetic risk (≤ 2 minor alleles in rs1535 and rs174448) as well as low DHA intake (< 200 mg DHA/d) had reduced milk DHA proportions. Increasing DHA intake only benefited milk of mothers with low but not high genetic risk. 

## Figures and Tables

**Table 1:** Characteristics of study participants ^1,2^.

Characteristics	All (n = 164)
**Maternal characteristics**	
Age (year)	32.7 ± 4.0
Height (cm)	160 ± 5
BMI (kg/m^2^) (before pregnancy)	23.6 ± 3.8
Parity	
Multiparous	76 (46.34)
Primiparous	88 (53.66)
Smoking during pregnancy	
No	160 (98.77)
Yes	2 (1.23)
Alcohol consumption during pregnancy	
No	151 (98.69)
Yes	2 (1.31)
Fish intake (serving/wk) ^3^	4.5 ± 2.4
DHA supplements	
No	108 (65.85)
Yes	56 (34.15)
Total DHA intake (mg/d) ^4^	143 ± 126
Low < 200 mg/d	120 (73.17)
High ≥ 200 mg/d	44 (26.83)
**Birth characteristics**	
Gestational age (wk)	38.6 ± 1.1
Gender	
Female	83 (51.23)
Male	79 (48.77)
Birth weight (g)	3135 ± 445
Birth length (cm)	50.2 ± 2.4

^1^ Data were presented as mean ± SD for continuous variables or n (%) for categorical variables. ^2^ BMI: body mass index. ^3^ One serving of fish = 35 g in raw. ^4^ Total docosahexaenoic acid DHA intake was calculated by sum of DHA intake from fish and supplements.

**Table 2:** Distributions of the two single nucleotide polymorphisms (SNPs) in study participants ^1,2,3^.

SNP	Gene	Minor/Major Allele (m/M)	Genotype			HWE *p*-Value	MAF (%)
			mm	mM	MM		
rs1535	*FADS2*	A/G	28 (17.07)	90 (54.88)	46 (28.05)	0.206	44.5
rs174448	*FADS2/3*	G/A	14 (8.54)	51 (31.10)	99 (60.37)	0.057	24.1

^1^ HWE: Hardy–Weinberg equilibrium; MAF: minor allele frequency. ^2^ MAFs of the Taiwan Biobank data: rs1535 A = 43.2% and rs174448 G = 27.7%. ^3^ MAFs of the CHS (Southern Han Chinese) in the 1000 Genomes data: rs1535 A = 41.4% and rs174448 G = 31.4%.

**Table 3:** Distributions of fatty acid compositions (percentage of total fatty acids identified) of human milk in study participants according to their genotypes ^1,2^.

Fatty Acid	All (n = 164)	SNP	Genotype			*p*-Value
			mm	mM	MM	
10:0	1.94 ± 0.63	rs1535	1.83 ± 0.63	1.91 ± 0.64	2.08 ± 0.63	0.180
		rs174448	2.03 ± 0.66	1.97 ± 0.69	1.92 ± 0.61	0.785
12:0	6.91 ± 2.51	rs1535	7.06 ± 2.65	6.74 ± 2.50	7.13 ± 2.45	0.650
		rs174448	7.38 ± 2.61	6.96 ± 2.54	6.81 ± 2.49	0.725
14:0	5.1 ± 1.9	rs1535	5.45 ± 2.23	5.03 ± 1.83	5.03 ± 1.84	0.583
		rs174448	5.51 ± 2.16	5.16 ± 1.86	5.02 ± 1.90	0.638
16:0	18.87 ± 2.36	rs1535	19.78 ± 2.8	18.81 ± 2.25	18.42 ± 2.16	0.050
		rs174448	19.07 ± 2.24	18.82 ± 2.44	18.86 ± 2.35	0.941
16:1	2.77 ± 0.61	rs1535	2.83 ± 0.63	2.74 ± 0.59	2.80 ± 0.64	0.724
		rs174448	2.69 ± 0.57	2.78 ± 0.54	2.78 ± 0.66	0.870
17:0	0.25 ± 0.42	rs1535	0.24 ± 0.08	0.28 ± 0.56	0.21 ± 0.07	0.672
		rs174448	0.20 ± 0.07	0.21 ± 0.07	0.28 ± 0.53	0.534
18:0	5.44 ± 1.2	rs1535	5.37 ± 1.31	5.41 ± 1.35	5.55 ± 0.78	0.786
		rs174448	5.63 ± 0.97	5.57 ± 1.17	5.35 ± 1.25	0.485
18:1	30.51 ± 7.81	rs1535	29.06 ± 8.36	30.39 ± 8.50	31.63 ± 5.77	0.381
		rs174448	29.61 ± 9.15	30.67 ± 7.74	30.56 ± 7.72	0.899
18:2n-6	22.8 ± 3.84	rs1535	22.39 ± 3.84	22.97 ± 4.07	22.71 ± 3.41	0.773
		rs174448	22.31 ± 3.09	22.63 ± 4.46	22.95 ± 3.61	0.788
18:3n-3	1.64 ± 4.97	rs1535	2.04 ± 5.52	1.88 ± 5.47	0.92 ± 3.37	0.508
		rs174448	2.15 ± 6.34	1.60 ± 4.85	1.58 ± 4.87	0.922
20:0	0.42 ± 0.35	rs1535	0.51 ± 0.38	0.41 ± 0.30	0.39 ± 0.40	0.341
		rs174448	0.44 ± 0.32	0.42 ± 0.40	0.42 ± 0.32	0.984
20:1n-9	0.51 ± 0.16	rs1535	0.59 ± 0.16^b^	0.52 ± 0.15^b^	0.43 ± 0.12^a^	<0.001
		rs174448	0.50 ± 0.22	0.49 ± 0.15	0.52 ± 0.15	0.596
20:2n-6	1.06 ± 0.29	rs1535	1.05 ± 0.24	1.07 ± 0.32	1.03 ± 0.25	0.707
		rs174448	0.99 ± 0.25	1.03 ± 0.25	1.08 ± 0.31	0.394
20:3n-6	0.26 ± 0.16	rs1535	0.28 ± 0.16	0.25 ± 0.16	0.26 ± 0.16	0.630
		rs174448	0.24 ± 0.14	0.22 ± 0.14	0.28 ± 0.17	0.127
20:4n-6	0.36 ± 0.44	rs1535	0.30 ± 0.23	0.42 ± 0.54	0.27 ± 0.22	0.151
		rs174448	0.33 ± 0.34	0.28 ± 0.25	0.40 ± 0.52	0.292
22:1n-9	0.84 ± 0.33	rs1535	0.85 ± 0.29	0.82 ± 0.34	0.88 ± 0.33	0.604
		rs174448	0.74 ± 0.25	0.81 ± 0.34	0.87 ± 0.33	0.243
22:6n-3	0.39 ± 0.26	rs1535	0.41 ± 0.33	0.39 ± 0.25	0.36 ± 0.22	0.683
		rs174448	0.36 ± 0.14	0.41 ± 0.35	0.38 ± 0.21	0.818

^1^ One-way ANOVA. When *p*-value < 0.05, a post-hoc Bonferroni test was used to detect differences among groups. ^2^ Values without a common superscript differed (*p* < 0.05).

**Table 4:** Multiple linear regression analysis of human milk DHA proportions (%) ^1.2^.

Variable	β (SE)	*p*-Value
Genetic risk (high vs. low)	−0.28 (0.13)	0.031
Total DHA intake (low vs. high)	−0.45 (0.17)	0.008
Gene–diet interaction	0.38 (0.17)	0.032
Age (year)	<0.01 (0.01)	0.457
BMI (kg/m^2^)	<0.01 (0.01)	0.980
Parity (multiparous vs. primiparous)	0.01 (0.04)	0.883
Gestational weeks	0.01 (0.02)	0.716

^1^ High genetic risk: minor allele number ≤ 2 in rs1535 and rs174448; low genetic risk: minor allele number = 3 in rs1535 and rs174448. ^2^ Low DHA intake: < 200 mg/d; high DHA intake: ≥ 200 mg/d.

## Supplementary Materials

The following are available online at https://www.mdpi.com/2072-6643/12/2/543/s1. Table S1 The sequences of the PCR primers and probes.
